# Using Social Media to Measure the Contribution of Red List Species to the Nature-Based Tourism Potential of African Protected Areas

**DOI:** 10.1371/journal.pone.0129785

**Published:** 2015-06-12

**Authors:** Louise Willemen, Andrew J. Cottam, Evangelia G. Drakou, Neil D. Burgess

**Affiliations:** 1 Department of Natural Resources, Faculty of Geo-Information Science and Earth Observation (ITC), University of Twente, Enschede, The Netherlands; 2 Institute for Environment and Sustainability, European Commission—Joint Research Centre, Ispra, Italy; 3 UNEP-World Conservation Monitoring Centre, Cambridge, United Kingdom; 4 Centre for Marcoecology, Evolution and Climate, Natural History Museum of Denmark, Copenhagen, Denmark; Institute of Agronomy, University of Lisbon, PORTUGAL

## Abstract

Cultural ecosystem services are defined by people’s perception of the environment, which make them hard to quantify systematically. Methods to describe cultural benefits from ecosystems typically include resource-demanding survey techniques, which are not suitable to assess cultural ecosystem services for large areas. In this paper we explore a method to quantify cultural benefits through the enjoyment of natured-based tourism, by assessing the potential tourism attractiveness of species for each protected area in Africa using the IUCN’s Red List of Threatened Species. We use the number of pictures of wildlife posted on a photo sharing website as a proxy for charisma, popularity, and ease of observation, as these factors combined are assumed to determine how attractive species are for the global wildlife tourist. Based on photo counts of 2473 African animals and plants, species that seem most attractive to nature-based tourism are the Lion, African Elephant and Leopard. Combining the photo counts with species range data, African protected areas with the highest potential to attract wildlife tourists based on attractive species occurrence were Samburu National Reserve in Kenya, Mukogodo Forest Reserve located just north of Mount Kenya, and Addo Elephant National Park in South-Africa. The proposed method requires only three data sources which are freely accessible and available online, which could make the proposed index tractable for large scale quantitative ecosystem service assessments. The index directly links species presence to the tourism potential of protected areas, making the connection between nature and human benefits explicit, but excludes other important contributing factors for tourism, such as accessibility and safety. This social media based index provides a broad understanding of those species that are popular globally; in many cases these are not the species of highest conservation concern.

## Introduction

Spatial assessments of ecosystem services (ES), aiming to support management of our natural environment, are increasingly common in science (e.g., [1, 2]) and in different realms of decision making [3–5]. Despite the overall progress in mapping, valuing and using information on ES for decision making, the assessments of non-material cultural benefits, such as experiences and cognitive development, that result from human-ecosystem relationships, have received little attention even though there is a growing demand for these services [6–10]. This is caused by the many challenges scientists encounter when assessing and locating cultural services, as these depend more than other ES on subjective stakeholder perceptions and values. Spatial assessments of cultural services can therefore rely less on generalizations, and instead require resource-demanding methods to capture the diverse preferences and perceptions of these intangible benefits [7, 9]. Studies that have quantified cultural ES are typically based on: economic valuations (including market values or willingness to pay), empirical assessments (such as determining spatial proxies for existing tourism sites), visitor counts, or small scale stakeholder surveys [6, 9, 11–13]. Large scale assessments at a cross-county or global level have used proxies of ecosystem characteristics to estimate provided cultural ES (e.g., [14, 15]).

One of the cultural ES that receives a large and growing demand from our industrializing society is the enjoyment of nature through recreation and tourism activities [16, 17]. Protected areas (PA) play a key role in nature-based tourism. PA are visited by tourists to enjoy different natural characteristics, such a geology (Yellowstone, USA), endemic natural vegetation (Páramo, the Andes), water bodies (Lake District, UK), or watching wildlife (African safaris). This non-consumptive form of nature-based tourism, can promote biodiversity conservation within the PA, if well managed [18]. Nature-based tourism is also believed to be an important, and growing, source of income and job generation in developing countries [13, 14, 19, 20]. Hernández-Morcillo et al. [21] showed in their review of cultural ES measures that in most studies no indicators on the underlying ecological state and processes are used to define cultural ES, but rather their end-values to humans. To be able to understand, and therefore better manage, ES flows, insight across the complete chain of biodiversity-ecosystem functioning- human well-being is required [9, 22]. Assessments of species that are most valuable for tourism, in monetary terms, have been reviewed and at the same time questioned by Catlin et al. [23]. Authors argue that monetary valuations of individual species are based on poor methods resulting in unreliable and misleading figures [23], a controversy which is more often stated for ES valuation studies [24]. Instead of commodifying species or describing tourism in infrastructure proxies (such as hotels, roads, facilities), we attempt to assess the attractiveness of a wide range of species as an indicator for tourism potential.

People find some species more attractive than others [10, 25]. To be able to quantify the attractiveness of species for wildlife tourism, we explored the use of photos of species posted on websites. We assume that species photo counts reflect key characteristics that determine species attractiveness including, species charisma, popularity, and ease to be recognized and ease to be observed in their habitat [25–28]. Social media is becoming a rich source of data on the public’s behaviour, ideas and values, and therefore valuable to assess subjective measures as cultural ES [29]. For example, a recent study on nature-based tourism found that the density of geotagged photos posted on the web corresponded well with the empirical information about where people travel to [30]. In another study, Casalegno et al. [11] used the number of people per square kilometer uploading photographs to a web platform as measure of aesthetic value.

In this paper we aim to construct an attractiveness index to assess the nature-based tourism potential for PAs, based on Red-Listed species occurring in an area. Our social media-derived index is intended to help understand how species contribute to the tourism potential of African PAs; an index that can be used—along with other tools- by conservationists, scientists and practitioners. Our index is based on minimal, freely available data and could therefore be used in large scale quantitative ES assessments when detailed surveys are not an option.

## Data and Methods

### Species and Protected Area data

We base our analysis on the species listed on IUCN’s Red List of Threatened Species [31]. As part of their assessment of global biodiversity, IUCN has developed species distribution maps for thousands of species of vertebrates and plants. Species distribution in this dataset is delineated by the known extent of occurrence, along with expert knowledge of habitat preferences and habitat suitability. The polygons depict the species distribution range, without implying that a species occurs everywhere within that polygon. Even though the IUCN spatial data does not give a precise description of species occurrence, it is the best available global dataset on where species are likely to occur. From the IUCN dataset we include all African species in our analyses that have spatial data and are categorized as extant, probably extant or possibly extant. This resulted in 6527 species from all IUCN listed taxonomic classes, including mammals, birds, reptiles, amphibians, fish, corals and a small number of insects, snails, ferns and flowing plants.

Information on African PAs was derived from the World Database of Protected Areas [32]. Since 1981 UNEP-WCMC, through its Protected Areas Programme, has been compiling and sharing global information on PAs, which range from strictly conserved National Parks to Community Reserves with human presence. From the entire WDPA dataset we selected all PAs for African countries, including Madagascar, for which PA boundaries are available. We included all PA management categories, but omitted very small parks with an area smaller than 100 ha and the PAs for which the park boundary location was unknown (represented by square polygons in WDPA). In total 5288 PAs were included in our assessment of which 280 are coastal PAs or fall within African territorial waters (12 nautical miles zone, approximately 22km).

### Species attractiveness

To derive a quantitative proxy on attractiveness of different species for tourism, including factors of popularity, charisma and ease of observation, we counted the number of photos of IUCN-listed species posted on the internet. The ease of wildlife to be observed includes aspects of visibility, diurnal versus nocturnal species, occurrence, and ease of identification. The number of online images for each species was counted based on a search on the binomial Latin species name in the Application Programming Interfaces (or APIs), which are available from the search engines Google, Bing, and Flickr. Flickr is the most established photo-sharing and management site and is now over 10 years old. APIs are freely available tools for searching web resources and allow for searching of images using all of the common search patterns and parameters. The domains in which the searches are made varies by provider; Google and Bing return the number of images that are available on the entire internet, whereas Flickr only returns the number of images on Flickr itself. Therefore the number of images for each species varies considerably between the different providers. Our main concern was to only include images in our analyses that actually depict the searched species. For this reason we had to abandon an original idea to search for images using the species’ common name in English, as this resulted in poor search results for species with common words in their English common name, such as ‘Red Kite’ or ‘Wild Cat’. After visual interpretation, we found that images resulting from a search for binomial names in Flickr showed the highest number of correct species depicted on the images compared to results from the Google or Bing APIs. Therefore, only the image counts resulting from the Flickr API search using binomial names are used in our analyses. We searched the contents of the photos as described in their label irrespectively of location where the picture was taken. We are aware that photos posted online might not have been shot in the wild, such as animals in zoos, but even in that case people only take photographs of the species they consider to be attractive.

Two corrections to the search results are made. First, one issue with searching the Flickr API is that the number of photos of species which have the same genus name as species name (e.g. *Gorilla gorilla*) return an unusually high number of images because of the way the search API interprets and uses the search term. In these cases the search was modified to search both on the binomial name and the English common name (e.g. Western Gorilla) to improve the relevance of the results.

Second, we attempted to exclude photos of species without a clear link to tourism, in particular images of very common species in gardens, pests, or domesticated pet species. These species have many images in Flickr but it is unlikely that for these common species people would travel to PAs to view them. For example the Mallard (*Anas platyrhynchos*), a common water bird in towns and cities with a range of occurrence extending to Africa, has 10 times as many posted images compared to the rare and threatened African Wild Dog (*Lycaon pictus*). To correct for these species which are unlikely to contribute to any tourism potential, we explored a number of different approaches: correcting for the total species range area (leaving out species with a very large range); correcting for the number of PAs that the species occurs in (leaving out species that occur in most PAs), and finally using the IUCN Red List status categories to remove all species that are classified as ‘Least Concern’ for conservation. We visually assessed the results of these options and decided that the best method to remove species with no clear link to tourism was by using the IUCN Categories. In our analysis of tourism potential we therefore leave out species with a ‘Least Concern’ status, even though this does exclude a number of popular tourist species such as Giraffe, Buffalo, Plains Zebra and Greater Flamingo. This correction therefore gives a higher importance to excluding species that are not relevant for tourism, at a cost of species which are known to be attractive to tourists. While we realize that this is not an ideal solution, this correction represents a pragmatic, transparent, repeatable and globally applicable method of prioritizing species that contribute to the nature-based tourism potential.

**Fig 1:**
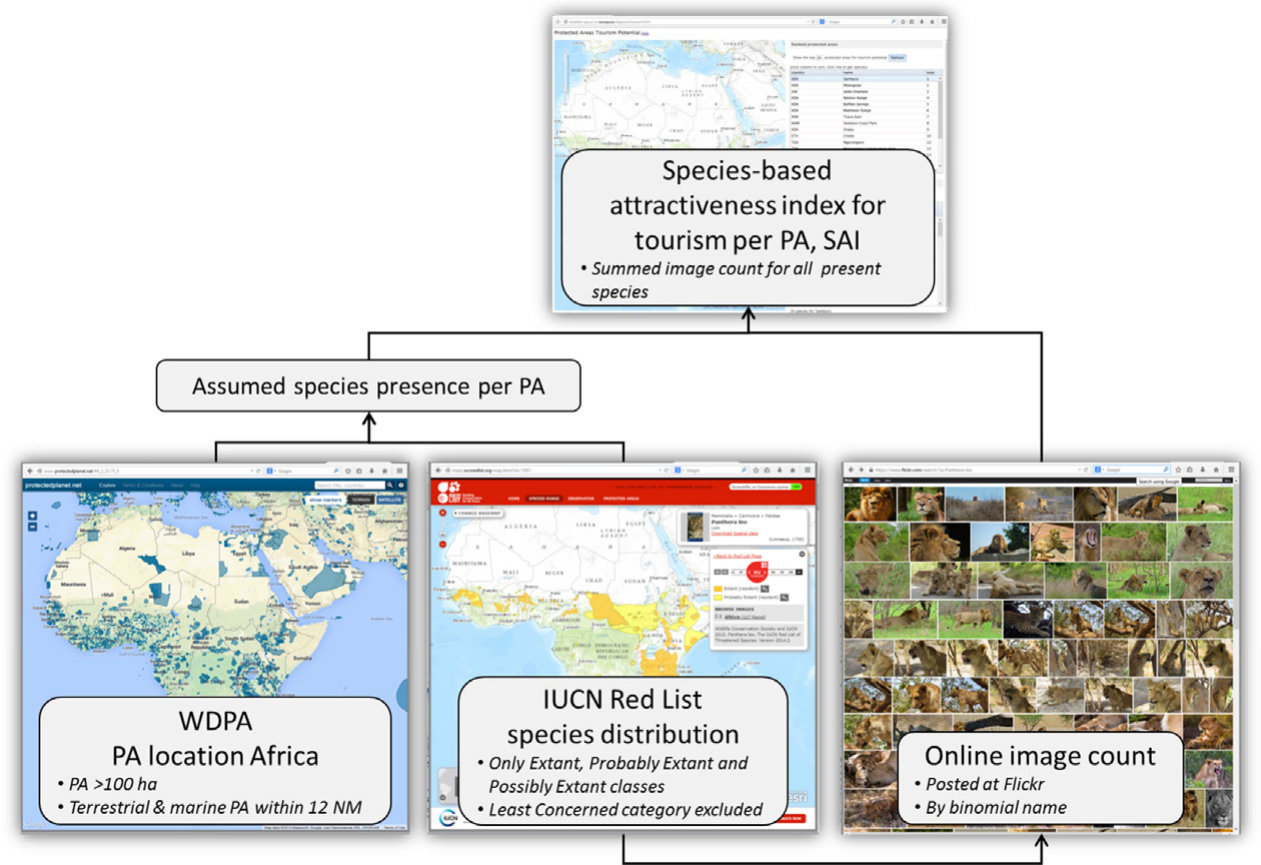
Use of freely accessible online data and filters to create the species-based tourism potential index for PAs in Africa.

The species attractiveness index (SAI) per PA is subsequently calculated by combining the IUCN Red List species range data, PA locations, and online image counts (Fig 1). The SAI for a PA was calculated as the sum of the image counts for each species that occurs in the PA based on the intersection between the IUCN Red List range data and the WDPA PA location. All species which have a range overlapping with a PA location were included; even if the area of intersection was very small. Per PA the SAI value is normalized to 0 to 1, following a simple non- transformative min-max normalization, making the results easier to present. For this transformation we subtracted the minimal value of SAI for all PAs in Africa from the SAI of that PA, and divided this by the range of SAI values for all African PAs. Fig 1 gives an overview of all the different data steps.

### Contribution of Red List species to nature-based tourism

We subsequently explored if species with different conservation statuses contribute differently to the total attractiveness for tourism of a PA. IUCN groups Red List species that occur in the wild by the following increasing ranks of conservation concern: Least Concern (LC), Near Threatened (NT), Vulnerable (VU), Endangered (EN) and Critically Endangered (CE). Evaluated species lacking assessment data are labeled by IUCN as Data Deficient (DD), whereas non-evaluated species are null in the dataset. Using a non-parametric Kruskal-Wallis test, we tested for differences between the Red List status and the number of posted images (with H0: no differences in distribution of photo counts are observed among the included Red List status classes CR, EN, VU, NT and DD).

There are a large variety of PAs. IUCN has developed guidelines to group PAs according to their (assigned) conservation management objectives (see Table 1). PA management designations by other international agencies include UNESCO Man and Biosphere Reserves, World Heritage Sites, Wetlands of International Importance through the Ramsar Convention and Special Protected Areas of Marine Importance through the Barcelona Convention. The WDPA lists IUCN management classes, if available, for each PA. All PAs with a designation by one of the other agencies are classified by IUCN as ‘Not Applicable’. We compared the summed photo counts per PA (the SAI), among these IUCN classes to test if our assessed tourism potential differs among these different PA management classes using a Kruskal-Wallis test (with H0: no differences in distribution of summed photo counts per PA are observed among IUCN classes)

**Table 1:** IUCN protected area management classes and objectives [33].

	Areas managed for:
I	Strict protection (1a Strict Nature Reserve, 1b Strict Wilderness area)
II	Ecosystem conservation and protection (i.e. National Park)
III	Conservation of natural features (i.e. Natural monument)
IV	Conservation through active management (i.e. Habitat species management area)
V	Landscape/seascape for conservation and recreation (i.e. protected landscape/seascape)
VI	Sustainable use of natural resources (i.e. managed resource protected area)

## Results

### Species attractiveness and tourism potential

**Fig 2:**
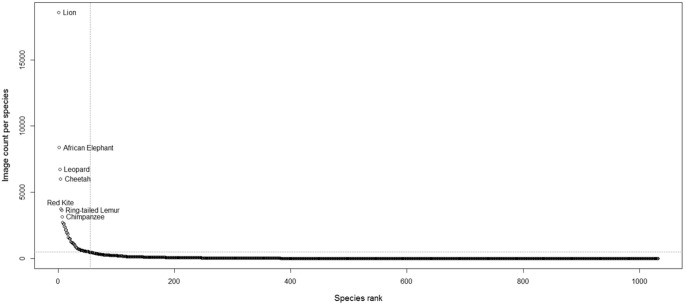
Species ranking and image counts per species. The highest 55 ranked species contribute to 72% of the total image counts.

Intersecting the selected IUCN Red List species range data with the location of the African PAs resulted in a list of 2473 species that are assumed to occur within these PAs. Most of these species are mammals (513), followed by bird species (365), amphibians (439), fish (253), reptiles (129), coral species (269) and the remaining 236 species of jellyfish, snails and plants. The Lion is by far the highest scoring species by photo counts, followed by the African Elephant, Leopard and Cheetah (Table 2). The first more uncommon species is the Red Kite (note, this search was performed on ‘*Milvus milvus*’ and its common name ‘Red Kite’ combined) occurring in northern Africa. In Fig 2 we plotted the number of images found in Flickr against the image count rank per species. The graph shows that after approximately 500 image counts, at the species ranked 55^th^ (the Saker Falcon), the graph starts to level. Meaning that around 2% of the searched species contribute 72% of the total found photos (104 366 images out of the total 143 461).

**Table 2:** The Top 25 of African species with the highest number of pictures posted on Flickr.

Binomial name	Common name	Photo count	Taxonomic Class	Red List status
*Panthera leo*	Lion	18574	Mammalia	VU
*Loxodonta africana*	African Elephant	8375	Mammalia	VU
*Panthera pardus*	Leopard	6737	Mammalia	NT
*Acinonyx jubatus*	Cheetah	5998	Mammalia	VU
*Milvus milvus*	Red Kite	3739	Aves	NT
*Lemur catta*	Ring-tailed Lemur	3666	Mammalia	NT
*Pan troglodytes*	Chimpanzee	3159	Mammalia	EN
*Hippopotamus amphibius*	Hippopotamus	2730	Mammalia	VU
*Limosa limosa*	Black-tailed Godwit	2635	Aves	NT
*Ceratotherium simum*	White Rhinoceros	2614	Mammalia	NT
*Numenius arquata*	Eurasian Curlew	2435	Aves	NT
*Spheniscus demersus*	African Penguin	2344	Aves	EN
*Negaprion brevirostris*	Lemon Shark	2165	Chondrichthyes	NT
*Physeter macrocephalus*	Sperm Whale	2152	Mammalia	VU
*Lycaon pictus*	African Wild Dog	1970	Mammalia	EN
*Balearica regulorum*	Grey Crowned-crane	1917	Aves	VU
*Pygoscelis papua*	Gentoo Penguin*	1835	Aves	NT
*Galeocerdo cuvier*	Tiger Shark	1594	Chondrichthyes	NT
*Oryctolagus cuniculus*	European Rabbit**	1535	Mammalia	NT
*Macaca sylvanus*	Barbary Macaque	1499	Mammalia	EN
*Falco naumanni*	Lesser Kestrel	1498	Aves	VU
*Equus grevyi*	Grevy's Zebra	1267	Mammalia	EN
*Diceros bicornis*	Black Rhinoceros	1229	Mammalia	CR
*Coracias garrulus*	European Roller	1212	Aves	NT
*Mandrillus sphinx*	Mandrill	1200	Mammalia	VU

* Occurs on an island in the sub-Antarctic Indian Ocean, an administrative part of South-Africa

** Occurs in northern Africa

The findings presented in Table 2 coincide with studies in South-Africa [26, 27] that showed that mega-herbivores and large carnivores were the most popular species, particularly among first-time and overseas visitors. The typical ‘big five’: elephant (*Loxodonta africana*), rhinos (*Ceratotherium simum* and *Diceros bicornis*), lion (*Panthera leo*) and leopard (*Panthera pardus*) also score high in our analysis. The buffalo (*Syncerus cafer*) is a species of Least Concern and was therefore excluded from our analysis.

**Fig 3:**
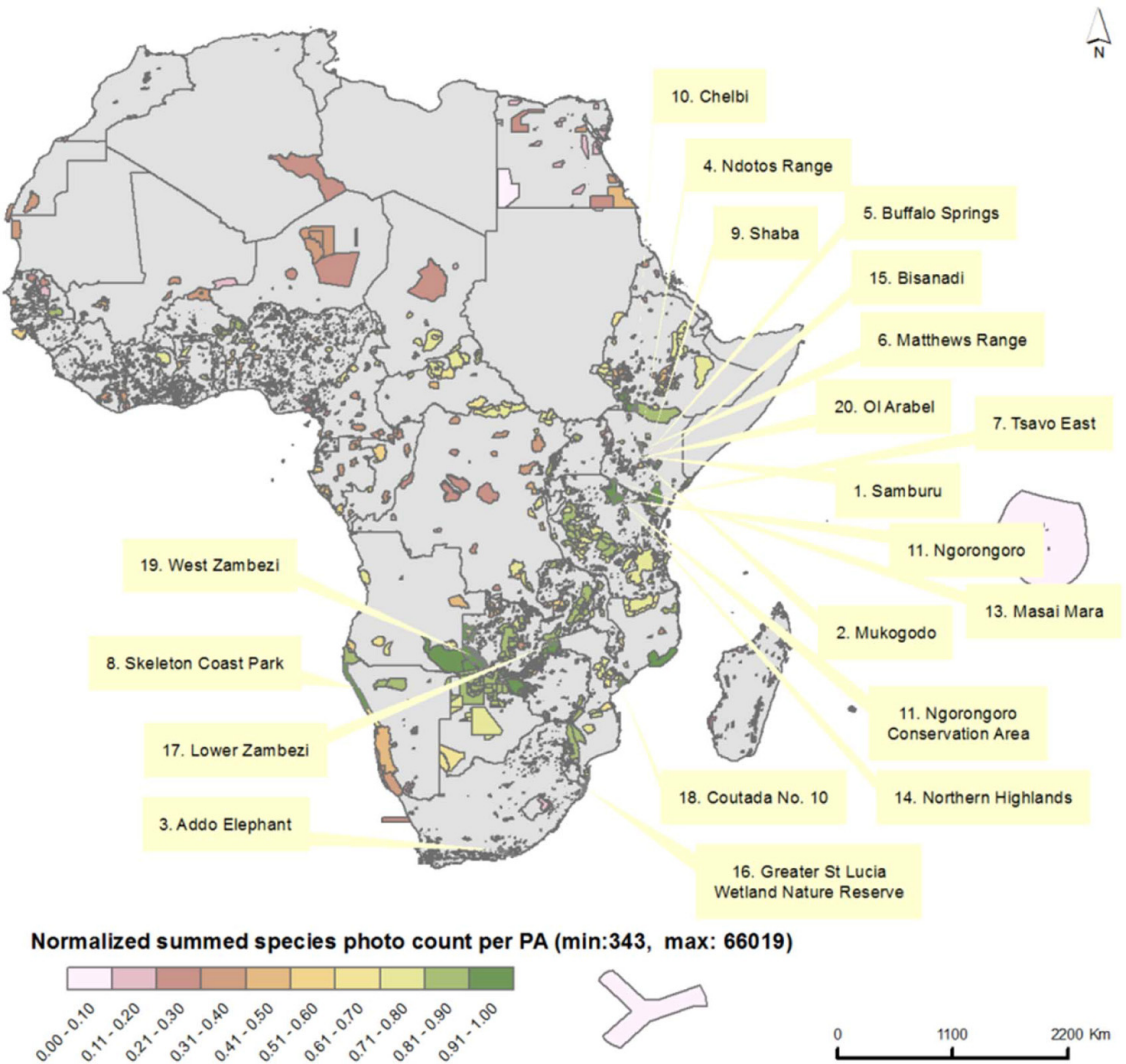
SAI as normalized sum of the photos counts per PA. The 20 highest ranked PAs by SAI are labelled.

For the 5288 African PA entries in the WDPA, the SAI values were calculated based on the photo count of the overlapping IUCN Red List species (Fig 1). Meaning that if PA overlays with 100 species, we summed the number of image counts from all 100 species to calculate the SAI for the PA. Fig 3 shows that the highest scoring PAs are located in east and southern Africa. This area supports the majority of African big cats, which give strong weight to the total score based on their high number of image counts (see Table 2, Fig 2).

All scores per PA and Flickr photos counts per species are published at this spatial user interface, http://andrewcottam.github.io/tourism_potential_africa/. Through this interface, users can search by PA, see what species are assumed to be present, and access information on the species Flickr counts and conservation status.

### Relating SAI, species richness and conservation strategies

We explored what type of species show up most often according to our search criteria in Flickr and therefore contribute most to a high SAI at the PA level. We found that the number of photos posted is positively correlated with the extent of species range, but only to a small extent (rho 0.41, or when 0 counts excluded rho 0.29, for p = 0.05). We also looked for associations between the IUCN Red List status and number of image posts. Based on a Kruskal-Wallis test, differences between the Red List status and the number of photos were found. The photo counts are heavily skewed towards zero, 1441 of the 2473 species were not found at all on Flickr. When excluding species for which no images were found (i.e. are not contributing the SAI), significantly more images were found for species categorized as Vulnerable and Near Threatened compared to the low scoring Data Deficient category (with p = 0.05). On average the Data Deficient species had the lowest number of photos posted (with an average count of 37 per species), whereas the Vulnerable (219) and Near Threatened (173) had the highest number of posted pictures on average. Note that these numbers are generated by a small number of highly popular species (Fig 2). However, an additional count revealed that species listed as of Least Concern for conservation, the class excluded from our tourist potential calculation, have the highest number of posts on Flickr (327 on average). These numbers show that the popularity of species at a photo sharing website does not follow the level of attention conservationists give to species to their degree of extinction threat.

**Fig 4:**
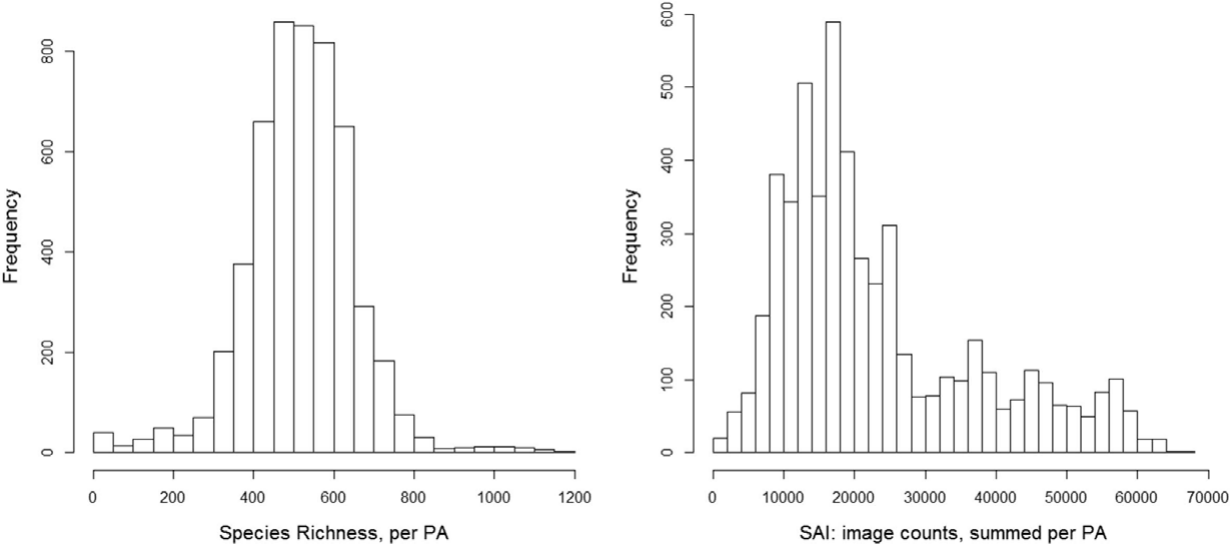
Frequency of richness classes and SAI for African protected areas; the effect of weighting attractive species of the PA richness with Flickr photo counts.

Our approach to defining species attractiveness can be seen as a species richness index weighted by species attractiveness for each PA. The left graph in Fig 4 shows the species richness per PA based in Red List range data and how often a PA with these richness values occurs in Africa (Fig 4, left). In the right graph we see that after weighting species presence with photo counts the distribution of values changes; whereas the richness in PAs follows a normal distribution, the SAI strongly differentiates PAs with a high tourism potential as a result of assigning higher values to more attractive species (excluding LC species).

**Fig 5:**
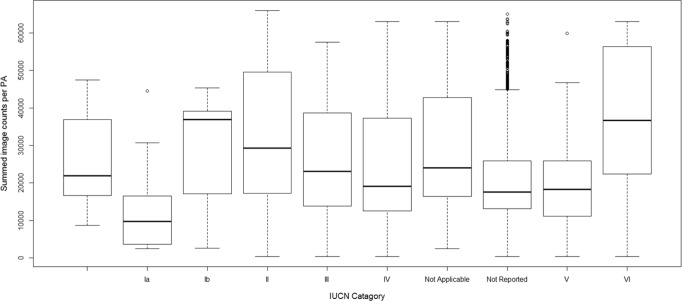
Differences in summed photo counts per IUCN management category. PAs managed as Strict Reserves, category Ia, score lowest on their total attractiveness for nature-based tourism. The boxes indicate the quantiles and the line the median. The Not Applicable class includes PAs managed as UNESCO Man and Biosphere Reserves, World Heritage Sites, Wetlands of International Importance through the Ramsar Convention and Special Protected Areas of Marine Importance through the Barcelona Convention.

When we compare our SAI results among the different IUCN classes for PAs, we find that the IUCN classes Ia (average of 11737 summed photos, or 0.17 when normalized) and VI (37629 photos on average, or 0.57 when normalized) are significantly (p = 0.05) different from the ‘not listed,’ class, used as reference, see Fig 5. Interestingly, the species-attractiveness index shows significantly (p = 0.05) lower values for strict nature reserves where tourism is not allowed (labeled as Ia) compared to the PAs that are grouped as areas for sustainable use (VI).

## Discussion, Validity & General Applicability

### A Species-based tourism attractiveness index and validation

We developed this method to explore if preferences for species could be included in a quantitative indicator to describe nature-based tourism potential at a large scale. One of the problems of producing metrics on tourism *potential* is that it is hard to discuss the validity of the results using figures based on observed *actual* tourism numbers, as differences could be caused by inaccurate assessments of the tourism potential or by other factors which influence tourism potential, such as security or PA accessibility. To explore the validity of our photo-count based index to assess nature-based tourism, we discuss our results at PA and species level.

For 69 of the PAs, we collected visitor data (for around the year 2009) and linearly regressed that data with the photo count-based SAIs, and found that that relation was not significant (p > 0.05, R-square of approximately zero). However, Tsavo East, Addo Elephant, Maasai Mara, and Zambezi PA which rank within the top 20 in the SAI list, also appear in the top 20 of highest reported visitor numbers in our limited dataset of 69 PA (in places 6, 8, 9, 19 respectively). We are well aware that actual tourism levels in an area are affected by a range of other factors besides the presence of attractive species [14]. For example, when we include minimal travel time from major cities (using the accessibility map by, [34]) and photo counts into the linear regression equation to explain the visitor number of the 69 PA, the R-square changes from approximately zero to 0.23, with travel time being the only significant factor (p <0.01). Meaning that *actual* attractiveness of park can be largely explained by its proximity to major cities (see also [14]). Safety and security for travelers also strongly impact the actual tourist numbers in Africa [35, 36] and if PA level information was available, these data would likely further explain the actual PA visitor numbers. Not including these factors in our species-based SAI explains that some well visited parks such as Amboseli NP in Kenya do not rank high based on their SAI value (Amboseli is ranked 86, see http://andrewcottam.github.io/tourism_potential_africa/).

**Fig 6:**
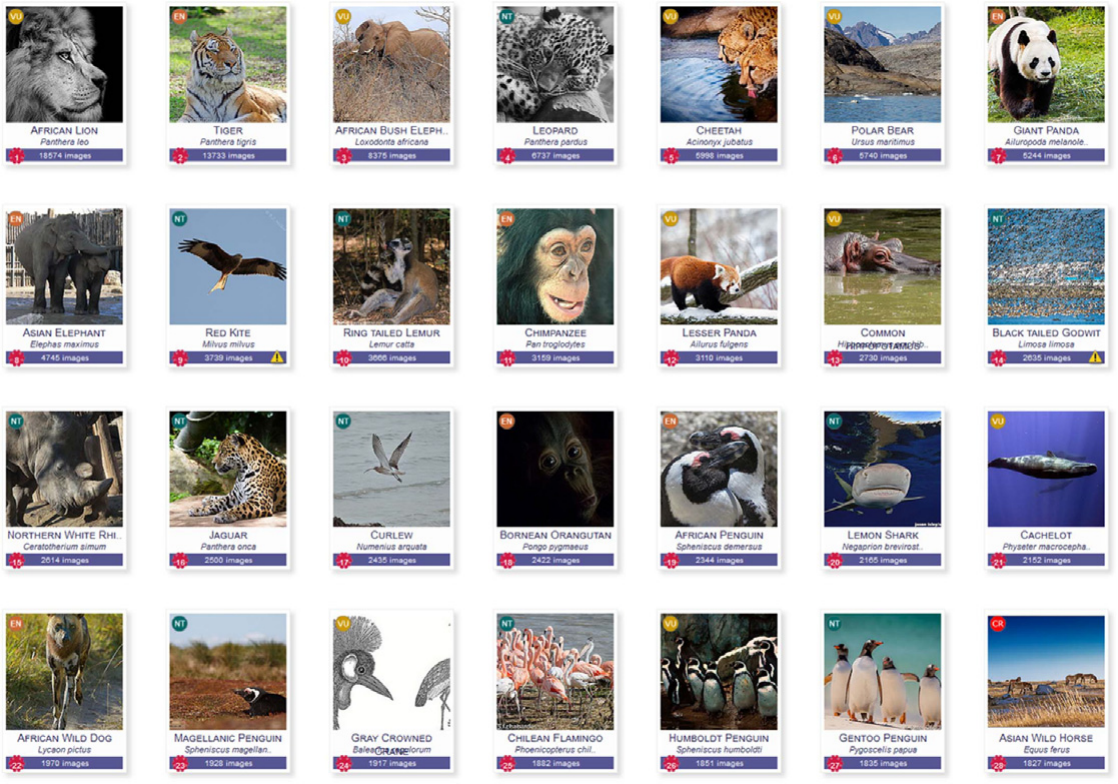
Top 28 of African and non-African Red List species with the highest number of photos posted in Flickr (species of Least Concern excluded).

To explore the validity of the use of species photo-counts in our SAI, we also counted photos posted in Flickr for all non-African species included in the IUCN Red list. That global species count resulted in high scores for widely known charismatic and iconic species, see Fig 6. The lion, tiger, African bush elephant, leopard, cheetah, polar bear, panda and Asian elephants lead the list (see the complete Top 100 http://andrewcottam.github.io/attractive_species_gallery/). This indicates a general validity of our explored method. Several earlier studies for South Africa found that these popular species are mostly attractive to international, first-time visitors to African PAs [26, 27, 37]. These studies highlight that African visitors and experienced wildlife viewers were more interested in bird and plant diversity, scenery, and rarer, less easily-observed, or less high-profile mammals.

The SAI of PA is calculated based on a summation of photo counts per species. We decided not to use any data transformations to calculate the SAI to 1) directly follow posted image counts as we could not justify any transformation choice, and 2) to keep ‘outlier’ effects. While smoothing data through transformations might reduce the impact of errors in photo counts, we are dealing with attractive species of which some have exponentially higher public attraction, i.e. these species would be outliers in the dataset. Our results show that only 2% of the studied species contribute 72% of the contributed photos, largely influencing the highest scoring PA. In the online interface we show the different SAI results when applying different transformations (log, sqrt, ranks, classes) and summation of species counts per PA (see http://andrewcottam.github.io/tourism_potential_africa/)

The input data used for the development of our index are the best available large scale datasets, but have known limitations, accuracies and uncertainties. For example, for many species maps in the IUCN database, the extent of occurrence that is shown is considerably larger than its realized range (area of occupancy), that leads to errors of commission when we intersect IUCN species range data with WDPA PA polygons. The degree to which this affects our results depends on the species—the more common species tend to have more generalized polygons than the more threatened species. Even though many very common species (often species of Least Concern for conservation) where excluded from our attractiveness assessment, we expect that our SAI is therefore over-assessing species richness within PA, even leading to situations where marine species ranges overlap with land (for example case for the PA ‘Coutada No. 9’ in Mozambique). Besides that, the SAI is based on binary range data; i.e. it does not include an assessment of the size of the population inside these boundaries, a factor that could also influence tourism attractiveness. The WDPA which was used to locate PAs also has known issues relating to areas boundaries, duplicates and missing IUCN management categories etc. Both the WDPA and IUCN Red List of Threatened Species are neither perfect or complete datasets, however both are the most comprehensive and scientifically rigorous information about the of state and distribution of species [38] and PAs [39]. Additionally our analysis aggregates large numbers of photos and species, which reduces the impact of misclassified Flickr images or incorrect range data.

Even though the use of social media to capture social preferences to assess a cultural ES is promising, issues about data quality, validation, and representativeness remain. The work presented in this paper on the use of online-posted photos to quantify species contributions to nature-based tourism could be seen as a first step in this. The index could be improved by more precise selection of images to able to also include species of LC typical of particular natural habitats and hence exclude the generalist and urban LC species.

### Insights for PA Management

If well-designed, equitably managed PAs could provide a powerful solution for maintaining ES, conserving biodiversity, and addressing the needs of human communities [18, 39]. In a recent review of IUCN World Heritage sites, tourism was cited most often, for 93% of the assessed PAs, as provided cultural benefit [20]. How could quantitative information on species attractiveness be used by PA managers? What would it imply knowing that a lion is ten times more attractive than a crowned crane? First of all we want to clarify that because of different spatial scales addressed, our quantified global SAI should not be used for individual PA management. So using social media in the first place gives insight into which species are most popular globally—but, which we found in many cases are not the species with highest global conservation concern.

However, it is worth mentioning that in our analysis strict reserves (a PA with IUCN category I) scored significantly lower on our tourism species-attractiveness index compared to the PAs that are categorized as areas for sustainable use (IUCN VI). This means that strict reserves have fewer and/or lower scoring attractive species compared to PAs with a human use. This finding highlights an opportunity to explore the tourism potential for areas that already have a sustainable use objective (IUCN VI), without interfering with strict biodiversity protection strategies in place in IUCN I areas. At the same time, this finding also highlights the challenge to PAs to design management strategies aimed at safeguarding both biodiversity and ES.

As our tourism-index by itself cannot be used to directly inform PA management, the index combined with information on visitors’ willingness to pay (WTP), access to the PAs, safety of the visited country, local infrastructure and others, might be interesting for processes influencing PA management at larger scale, such as targeted fundraising based on the species attractiveness, or adjusted conservation strategies for less attractive species [40]. Online media applications could play a role in this. For example, the WCMC’s Protected Planet website overlays IUCN species distribution data with PA boundaries and show their images. For specific fundraising or attracting visitors, these species could be listed by order of our attractiveness index.

## Conclusions

The presented species-attractiveness index contributes to the quantification of the potential for natured-based tourism in Africa, using the photos posted in social media to weight individual species attractiveness. Social media is becoming a rich source of data on the public’s behaviour, ideas and values, and therefore a new and promising way to assess subjective measures such as cultural ES. With this paper we hope to promote debate and move this area of work forward. The proposed method uses three global data sources which are freely accessible and available online which makes the index attractive for large scale quantitative ES assessments, including global and continental level ES accounting and ES modeling. The index links species presence to the tourism potential of PAs, making the connection between nature and human benefits explicit. Yet it still excludes other important contributing factors for realized tourism such as accessibility and tourist facilities. Using a social media based index in the first place gives insight into what species are most popular globally; these are in many cases not the species with highest conservation concern. This finding highlights the challenge to PAs to design management strategies aimed at safeguarding biodiversity and ES.
